# Atypical Carcinoid Tumor of the Mediastinum Presenting as Cushing’s Syndrome in an Otherwise Healthy Young Male

**DOI:** 10.7759/cureus.14940

**Published:** 2021-05-10

**Authors:** Ian Landry, Luis A Medina Mora, Raheel Siddiqui, Taisiya Tumarinson, David M Reich

**Affiliations:** 1 Medicine, Icahn School of Medicine, Queens Hospital Center, New York City, USA

**Keywords:** cushing's disease, neuroendocrine tumor, ectopic acth, anterior mediastinal mass, atypical carcinoid tumor

## Abstract

Primary neuroendocrine tumors (NETs) are rare types of malignancies that can have a variety of presentations due to the ubiquitous distribution of neuroendocrine cells within the body. While mediastinal masses are not uncommon, NETs arising from the anterior mediastinum are rare and often originate from the thymus gland. A subset of NETs, atypical carcinoids, are more commonly seen in the lungs or gastrointestinal organs and often present with endocrine syndromes, chiefly Cushing’s syndrome. The behavior of atypical carcinoid tumors within the mediastinum is often aggressive and clinical presentations vary widely. In this report, we describe a case of an atypical carcinoid tumor within the anterior mediastinum in an otherwise healthy young male with signs and symptoms of Cushing’s syndrome.

## Introduction

Primary neuroendocrine tumors (NETs) derive from neuroendocrine cells, which are present in many organ systems, thus, these tumors may arise in various anatomical locations. While NETs are considered very rare, their reported incidence is increasing. Overall, it is estimated more than 12,000 people in the United States are diagnosed with NETs each year; with approximately 175,000 people currently living with the diagnosis [1]. Neuroendocrine tumors of the thymus (NETT) are very rare and aggressive compared to pulmonary and GI tract neuroendocrine tumors [2]. NETT accounts for 2-4% of the cases of anterior mediastinal mass [3]. 

Overall, neuroendocrine neoplasms are classified into well-differentiated NETs and poorly differentiated neuroendocrine carcinomas (NEC) [3]. The NETs are graded in three tiers (based on the mitotic count, Ki-67 labeling index, and presence or absence of necrosis) as G1, G2, and G3 corresponding to low-grade, intermediate-grade, and high-grade, respectively. Lung and thymic typical carcinoids inherently reflect G1, and atypical carcinoids reflect G2. Neuroendocrine carcinomas (NEC) include large-cell neuroendocrine carcinomas and small-cell carcinomas. It is not necessary to grade NEC as these tumors are always high-grade [3,4]. These distinctions are important, as different morphologies appreciate significant survival differences [5]. Atypical carcinoid tumors typically have an aggressive clinical course exhibited by local recurrence or distant metastasis in approximately 20-30% of patients [6,7]. This aggressive behavior correlates well with histologic grade, which is itself directly proportional to the degree of differentiation. We report a case of severe, treatment-resistant ectopic Cushing’s syndrome in a patient with a large, atypical carcinoid tumor secreting ectopic adrenocorticotropic hormone (ACTH). This tumor was successfully treated by management of the hypercortisolism with ketoconazole and metyrapone, followed by resection of the mediastinal mass.

This article was submitted and presented to the Journal of Endocrine Society at ENDO 2021 (2021; 5:991-992).

## Case presentation

A 38-year-old male with a past medical history of untreated, mild depression diagnosed several years ago was admitted for the subacute presentation of nonspecific fatigue and weakness associated with nausea and subjective weight gain over several weeks. Physical examination was significant for conjunctival pallor and decreased breath sounds on the left anterior chest. Initial laboratory findings were significant for K = 2.4 mmol/L (reference range: 3.5-5.1), Cl = 95 mmol/L (reference range: 98-108), HCO_3_ = 41 mmol/L (reference range: 22-29), and Na 145 mmol/L (reference range 136-145). He was noted to have WBC 12.0 x 10^3^/mcL (reference range: 4.8-10.8) and lactate 2.1 mmol/L (reference range: 0.5-2.0). Initial vitals included temperature 98.6F, heart rate 79, respiratory rate 16, blood pressure 129/78 mm Hg with oxygen saturation 98% on room air. 

**Figure 1:**
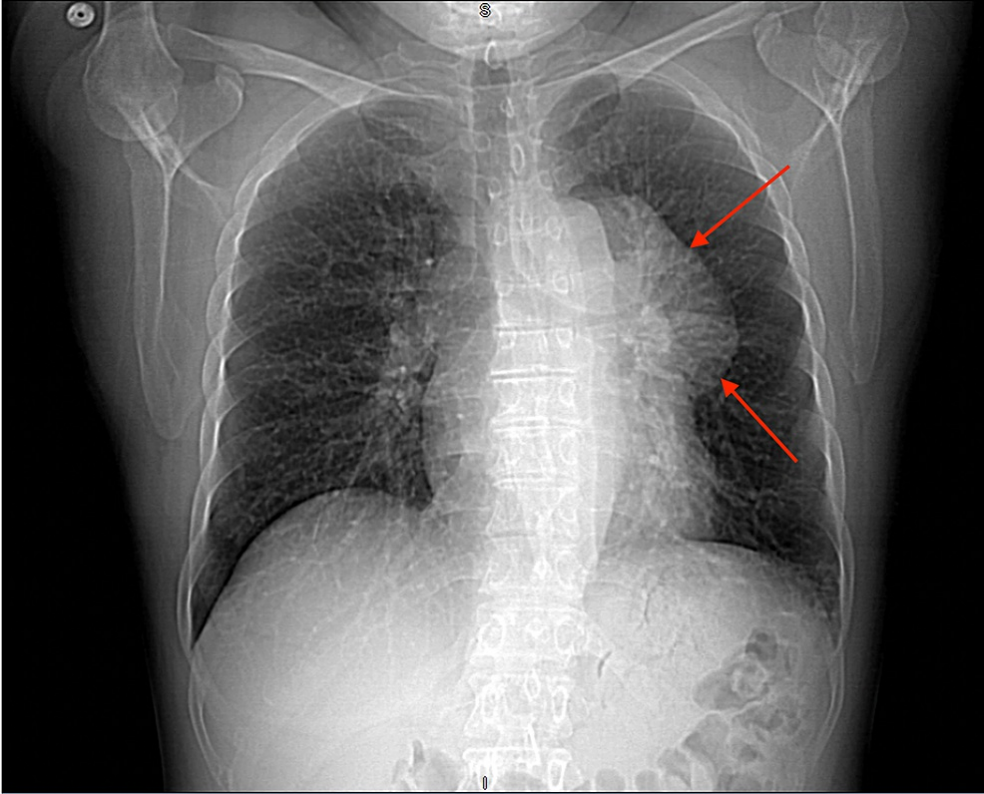
Chest X-ray, anterior view, initial presentation showing anterior mediastinal mass which extends into the left hilum

**Figure 2:**
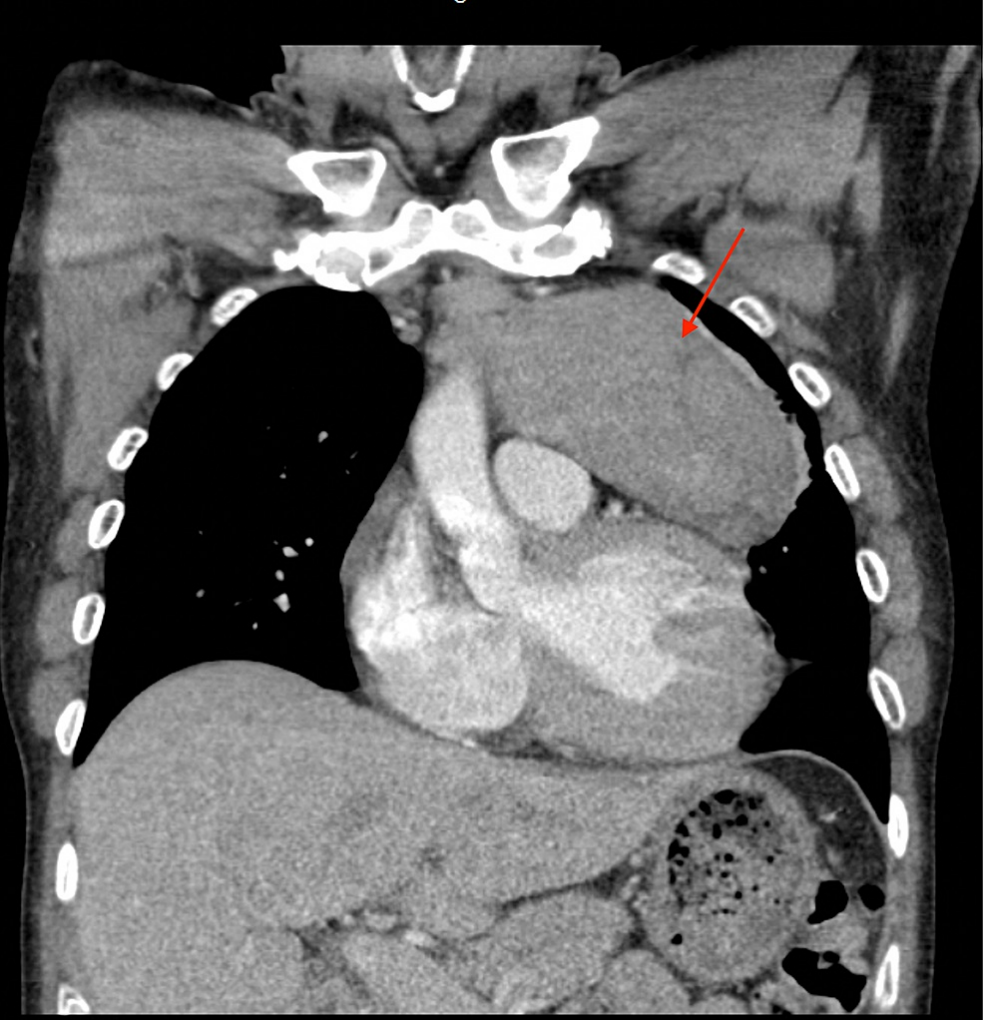
Chest-CT with contrast, coronal view, 10.3 cm anterior mediastinal mass with two adjacent enlarged mediastinal lymph nodes

**Figure 3:**
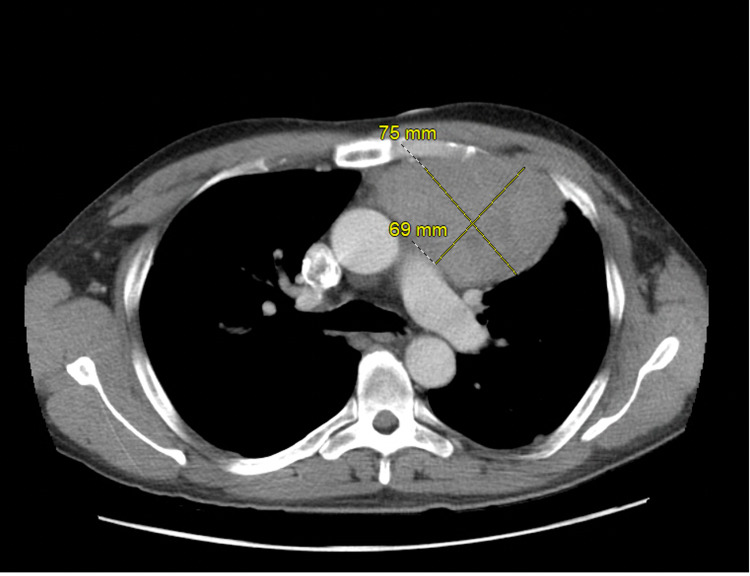
Chest-CT with contrast, axial view

**Figure 4:**
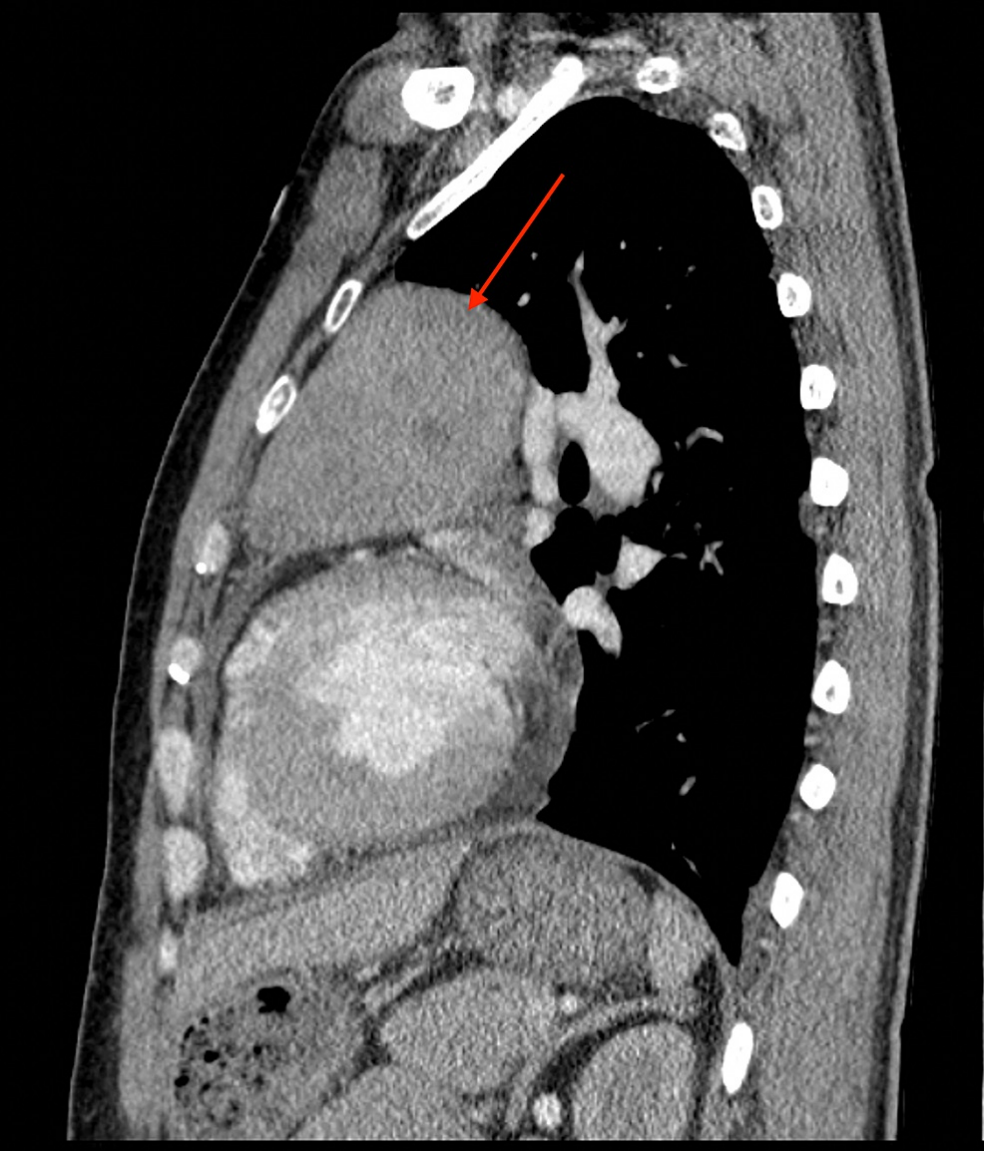
Chest-CT with contrast, sagittal view

Chest x-ray (CXR) showed a prominent anterior mediastinal mass extending into the region of the left hilum (Figure 1). Subsequent chest CT revealed a mildly heterogeneous anterior mediastinal mass measuring approximately 9.0 x 10.3 x 6.1 cm, extending to the left and exerting mild mass effect on the left upper lobe of the lung (Figures 2-4). There were two enlarged mediastinal lymph nodes adjacent to this mass, without hilar or axillary lymphadenopathy. Initial workup for common mediastinal masses, including testicular examination with ultrasound and tumor markers for germ cell neoplasia, returned negative. Thyroid function testing was normal. Acetylcholine receptor antibodies were negative. Morning cortisol was elevated at 47.7 ug/dL (reference range: 6.0-21.0), ACTH 281 pg/mL (reference range: 7.2-63.3) with suppressed aldosterone <3.0 ng/dL (reference range: <23.2) and renin <2.1 pg/mL (reference range: <33.2). The morning serum cortisol after 8 mg dexamethasone at midnight, was 58.7 mcg/dL. Chromogranin A was elevated at 423 ng/mL (reference range: <93) as well as 5-hydroxyindolacetic acid at 2,950 mg/day (reference range <6.0 mg/day). 

**Figure 5:**
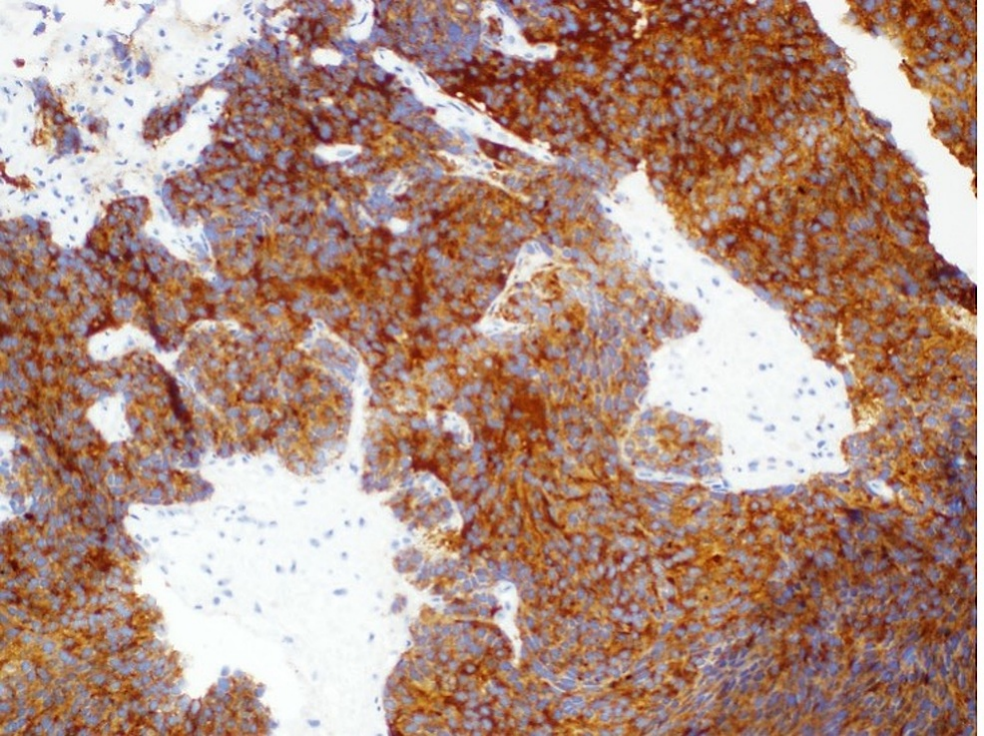
Histology of biopsy showing specimen was positive for synaptophysin

**Figure 6:**
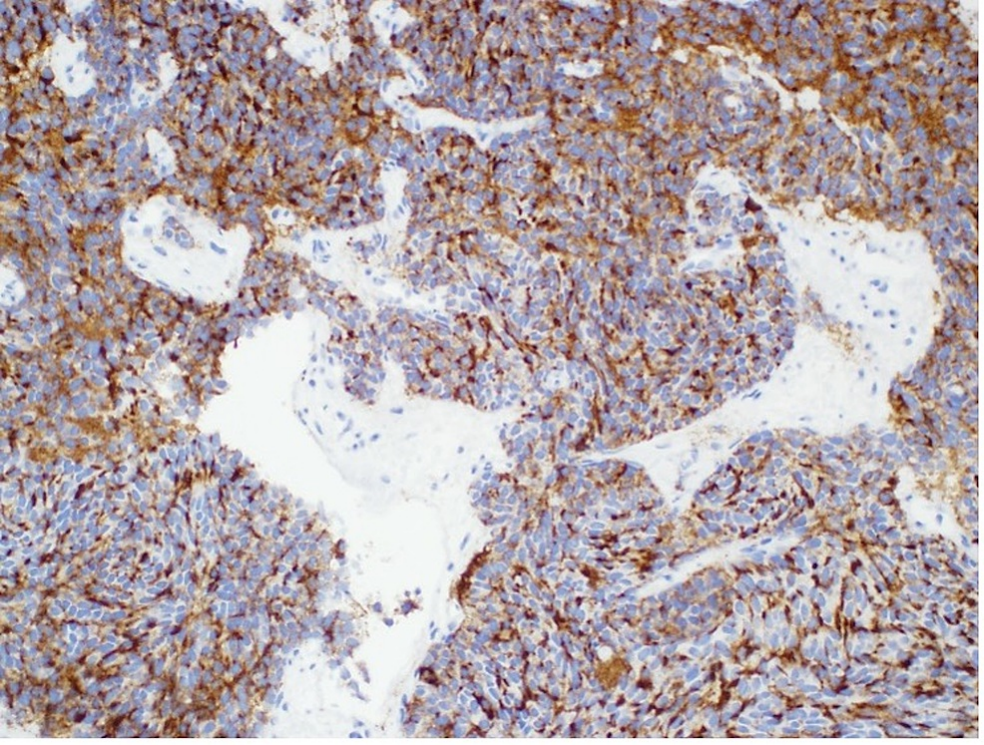
Histology of biopsy showing specimen was positive for chromogranin

**Figure 7:**
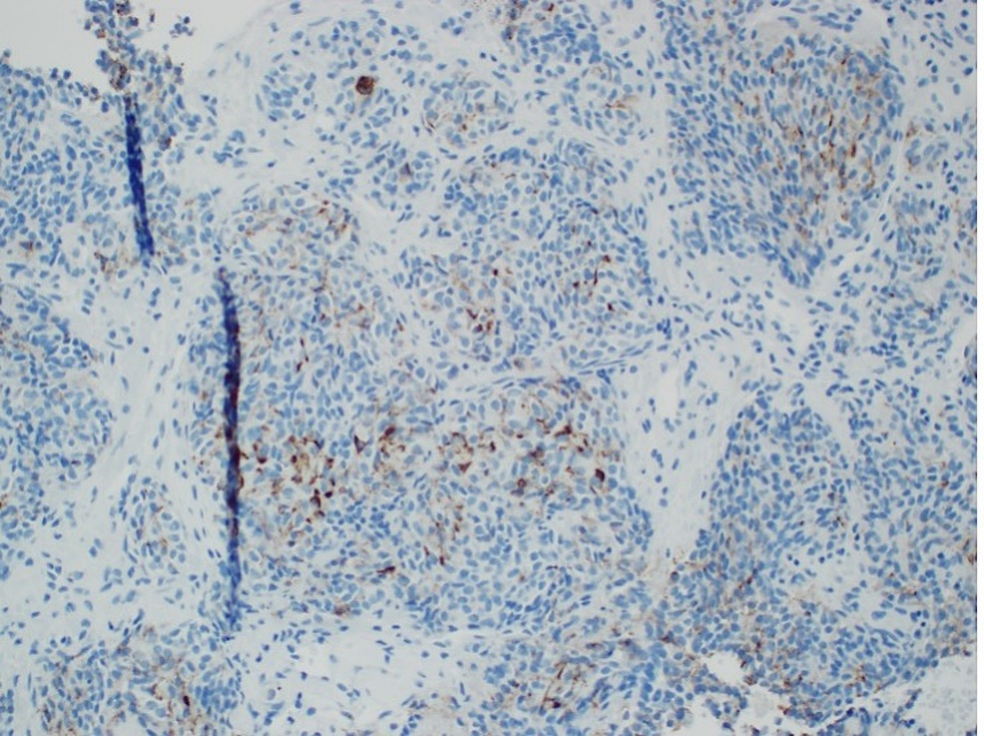
Histology of biopsy showing specimen was partially positive for ACTH ACTH: adrenocorticotropic hormone

**Figure 8:**
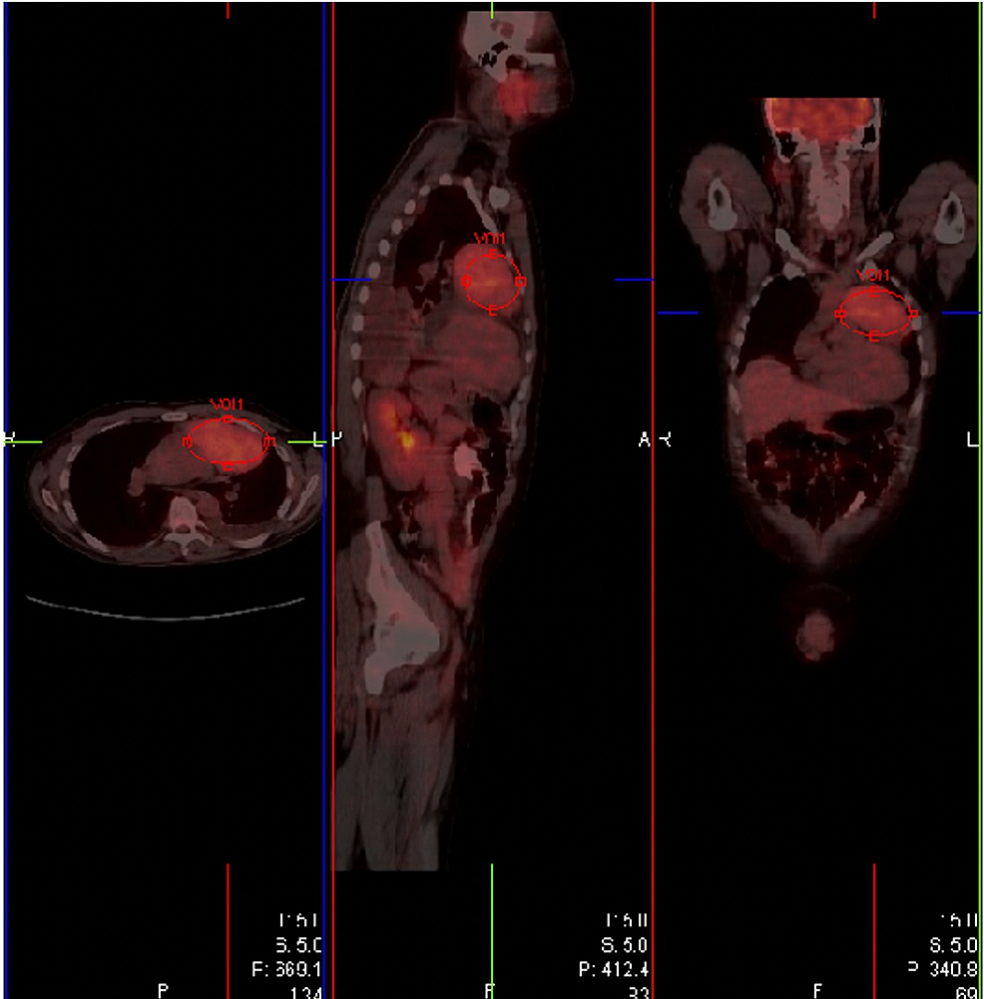
PET-CT showing heterogenous increased uptake localized to an approximately 9.5 x 7 cm soft tissue mass in the anterior mediastinum. SUV max 4.3. SUV: standardized uptake value

An Interventional radiology-guided biopsy of the mediastinal mass returned positive for a neuroendocrine tumor, grade 2, atypical carcinoid, rather than thymoma. Spindle cells were positive for cytokeratin AE1/E3, CAM 5.2 and negative for CK7. The tumor cells were also positive for synaptophysin (Figure 5), chromogranin (Figure 6), ACTH (partial) (Figure 7), and Ki-67 3-5%. The patient was started on spironolactone for hypokalemia and metabolic alkalosis. Initial metastatic workup, including CT of the abdomen/pelvis, MRI brain, and PET-CT (mass with max standardized uptake value 4.3; see Figure 8) were negative for metastatic disease. The patient was subsequently transferred to another institution for surgical evaluation, where he was started on metyrapone and ketoconazole, with a subsequent decrease in his cortisol level. While waiting for operative management, the patient developed persistent fevers and found to be in an immunocompromised state (absolute cluster of differentiation 4 {CD4} 98, immunoglobulin G {IgG} 510, immunoglobulin M {IgM} 33) with negative workup for HIV and hepatitis viruses. He underwent CT chest/abdomen/pelvis with findings of multifocal areas of ground-glass opacities suspicious for pneumonia. He was incidentally found to have a right femoral lesion, not previously noted on PET CT. MRI of the femur was suspicious for avascular necrosis of the femur. Given the patient’s high risk of venous thromboembolism due to underlying malignancy, infection, and hemodynamic instability he was transferred to the intensive care unit for adrenal suppression to goal cortisol <20. 

The patient underwent resection of the anterior mediastinal mass and thymectomy en bloc with pericardium 30 days after admission. During surgery, the patient received 50 mg hydrocortisone, with long-term dosing afterward. Surgical pathology returned positive for atypical carcinoid tumor with zero of three lymph nodes positive for the disease. During his postoperative period, the patient had persistent tachycardia with a heart rate up to 140s. CT-chest angiography revealed no evidence of pulmonary embolism but again revealed bilateral consolidations indicative of pneumonia. As a result of his operation, the patient developed pericarditis without pericardial effusion, which was thought to be in the setting of recent pericardial patch placement. For the management of his acute pericarditis, he received nonsteroidal anti-inflammatory drugs (NSAIDs) and colchicine. He was stabilized and discharged on hydrocortisone with planned follow-up with CT surgery, orthopedic surgery, and medical oncology. As surgical pathology was notable for margins <1mm of distance from the tumor, adjuvant radiation was considered but shared decision making between the radiation oncology team and the patient led to a decision for active surveillance. A follow-up echocardiogram was significant for signs of early tamponade (right ventricular diastolic collapse, left atrial free wall inversion, and right atrial free wall inversion). The patient underwent pericardiocentesis with cytology returning negative for malignant cells. Follow-up imaging at three months post-op with chest CT and Octreoscan showed mild, focal uptake at the site of midline sternotomy with linear uptake in the soft tissues of the superior margin of the pericardium, which was more suggestive of residual disease. As the patient was tapered off of corticosteroids, ACTH levels were frequently monitored for signs of recurrence. Initially, the levels increased to 73.9 pg/mL (reference range: 7.2-63.3), but subsequent values have normalized. At the last follow-up, the patient has resumed working and is under active surveillance.

## Discussion

Neuroendocrine tumors can arise from multiple organs and tissues. While they are more commonly seen as gastrointestinal or pulmonary tumors, they can present in the mediastinum. Atypical mediastinal neuroendocrine tumors have been described in the literature but remain a source of controversy as to their true origin and classification [3,6]. Mediastinal atypical carcinoid tumors have been observed to have aggressive behavior and exhibit features of pulmonary carcinoid tumors, with prevalent endocrinopathies [8,9]. 

We describe a case of a primary neuroendocrine tumor found in the anterior mediastinum of a healthy young male. At presentation, his differential diagnosis included the classic “four Ts” of an anterior mediastinal mass: thymoma, teratoma, thyroid carcinoma, and “terrible” lymphoma. A diagnosis of teratoma was excluded based on negative testicular ultrasound and tumor markers. Thyroid abnormalities were less likely due to normal thyroid function testing and lack of evidence for hyper/hypothyroidism. Thymoma vs. lymphoma were strong considerations. The patient complained of nonspecific weakness and fatigue, worrisome for possible myasthenia gravis as a sequela of thymoma or paraneoplastic syndrome but ruled out by negative antibody testing. Interventional radiology (IR)-guided biopsy with subsequent chromogranin A elevation to 423 ng/mL and severely elevated 5-hydroxyindoleacetic acid (5-HIAA) level of 2,950 mg/day favored a diagnosis of neuroendocrine tumor. 

The hypercortisolemia in Cushing’s syndrome has several etiologies, including: (1) ACTH-producing pituitary tumors (Cushing’s disease), (2) ectopic ACTH production by non-pituitary tumors, (3) excessive cortisol secretion from adrenal adenoma/carcinoma, and occasionally (4) ACTH-independent cortisol secretion by adrenal cortical hyperplasia. While this clinical presentation was not the typical picture associated with Cushing’s syndrome (chiefly, abdominal striae, acne, excessive weight gain, abnormal fat distribution, and frequent bruising) close inspection of his symptomatology and laboratory findings elucidated Cushingoid features. Our patient’s nonspecific weakness, subjective weight gain, and severe electrolyte abnormalities are consistent with elevated ectopic production of ACTH, which increases serum cortisol. The patient's morning cortisol levels were found to be severely elevated, with a maximum value of 61.2 ug/dL (reference range: 6-21). Urinary cortisol levels were 8,041 mcg/24 hours (reference range: 3.5-45). ACTH reached a peak value of 357 pg/mL (reference range: 7.2-63.3), which was not suppressed by high-dose dexamethasone testing, indicative of ectopic production. 

Glucocorticoids are known to have activity at mineralocorticoid receptors (MR) within the target cells of aldosterone. Under physiologic levels, aldosterone competes with glucocorticoids for the MR; inactivation of cortisol by the enzyme 11-β-hydroxysteroid dehydrogenase 2 (11B-HSD2) (present in most tissues) limits the activity of cortisol [10]. However, in cases of ectopic production of ACTH leading to hypercortisolemia, activation of the MR can lead to significant electrolyte disturbances, including hypernatremia, hypokalemia, and resultant metabolic alkalosis. 

The earliest reports of Cushing's syndrome noted an association between immunosuppression and glucocorticoid levels [11]. In many reports, multiple opportunistic infections are evident in patients due to widespread immunosuppression. Our patient’s clinical course was complicated by notable decreases in T-cell count (CD4 specifically) and decreased IgM and IgG without infectious etiology, which lead to recurrent febrile illness, suggestive of cortisol-induced immunosuppression. 

Avascular necrosis of the femur has been known to present in younger adults as a result of chronic exogenous corticosteroid use. Glucocorticoids have been shown to have a dose-dependent effect on the skeletal system which over time leads to bone loss and fracture. Studies evaluating this pathophysiological mechanism have suggested that inhibition of the mammalian target of rapamycin (mTOR) signaling in response to glucocorticoid stimulation leads to induction of apoptosis in femoral head progenitor cells [12]. 

## Conclusions

Primary neuroendocrine tumors of the mediastinum are rare malignancies. Current literature suggests that these tumors often express a more aggressive behavior and are associated with endocrinopathies. We presented a case of a young, healthy male who presented with nonspecific symptoms, but who had severe metabolic, immunologic, and orthopedic complications which can be explained by his excessive glucocorticoid burden. While many patients with Cushing's syndrome may present in a more typical fashion, high clinical suspicion must be considered in all patients who present with nonspecific signs of fatigue, weakness, and abnormal chemistry profile in the setting of a mediastinal mass. Survival depends largely on the patient's baseline function and tumor behavior; however, early recognition of these malignancies can lead to better outcomes and prevention of Cushingoid complications.
